# Ligand-Based Virtual Screening, Molecular Docking, and Molecular Dynamic Simulations of New β-Estrogen Receptor Activators with Potential for Pharmacological Obesity Treatment

**DOI:** 10.3390/molecules28114389

**Published:** 2023-05-27

**Authors:** Domingo Méndez-Álvarez, Maria F. Torres-Rojas, Edgar E. Lara-Ramirez, Laurence A. Marchat, Gildardo Rivera

**Affiliations:** 1 Laboratorio de Biotecnología Farmacéutica, Centro de Biotecnología Genómica, Instituto Politécnico Nacional, Reynosa 88710, Mexico; doomadv@hotmail.com (D.M.-Á.); elarar0700@hotmail.com (E.E.L.-R.); 2 Laboratorio de Biomedicina Molecular 2, Escuela Nacional de Medicina y Homeopatía, Instituto Politécnico Nacional, Ciudad de México 07320, Mexico; fernandatr.ipn@gmail.com (M.F.T.-R.); lmarchat@gmail.com (L.A.M.)

**Keywords:** ligand-based virtual screening, molecular docking, molecular dynamics, estrogen receptor beta, obesity

## Abstract

Obesity is a pandemic and a serious health problem in developed and undeveloped countries. Activation of estrogen receptor beta (ERβ) has been shown to promote weight loss without modifying caloric intake, making it an attractive target for developing new drugs against obesity. This work aimed to predict new small molecules as potential ERβ activators. A ligand-based virtual screening of the ZINC15, PubChem, and Molport databases by substructure and similarity was carried out using the three-dimensional organization of known ligands as a reference. A molecular docking screening of FDA-approved drugs was also conducted as a repositioning strategy. Finally, selected compounds were evaluated by molecular dynamic simulations. Compounds **1** (−24.27 ± 0.34 kcal/mol), **2** (−23.33 ± 0.3 kcal/mol), and **6** (−29.55 ± 0.51 kcal/mol) showed the best stability on the active site in complex with ERβ with an RMSD < 3.3 Å. RMSF analysis showed that these compounds do not affect the fluctuation of the Cα of ERβ nor the compactness according to the radius of gyration. Finally, an in silico evaluation of ADMET showed they are safe molecules. These results suggest that new ERβ ligands could be promising molecules for obesity control.

## 1. Introduction

The most widespread health problem in the world is obesity. It is a condition characterized by excess adipose tissue, an associated increase in the size and number of white adipocytes, and a body mass index (BMI) ≥ 30 kg/m^2^ [1]. Obesity triggers cardiovascular diseases, diabetes, and some types of cancer, which are the main causes of morbidity/mortality worldwide, musculoskeletal disorders, and others [2].

A recent study of data from 195 cities since 1990 showed that obesity has continuously increased, affecting more than 107.7 million children and 603.7 million adults [3]. Different methods have been used to reduce and maintain an adequate BMI (<25 kg/m^2^). A low-calorie diet and increasing physical activity are the primary strategies to balance energy intake and expenditure; however, people have problems maintaining this healthy lifestyle [4]. Drugs are another option to control body weight and avoid obesity-associated diseases [5,6]. Rimonabant, a CB1 cannabinoid receptor antagonist, emerged as a new option for obesity; however, this drug cause central side effects; therefore, new strategies are explored to improve the biological effects and reduce their adverse effects [7,8]. Recently, semaglutide, a second GLP1R agonist, was approved by the FDA [9]. In general, all drugs approved for the pharmacological treatment of obesity have several side effects, such as gas retention, tachycardia, and association with thyroid cancer, which strongly limit their use. Likewise, bariatric surgery is a high-cost, risky procedure available to a limited number of patients with morbid obesity [5,6]. Therefore, developing new therapeutic strategies for obesity is a main concern.

Estrogen receptors (ER) are ligand-dependent transcription factors that interact with other nuclear transcription factors, crosstalk with growth factor systems, and are associated with modulators [10]. The most prominent ERs, ER alpha (ERα) and ER beta (ERβ), have a similar molecular organization. Both ERs contain the N-terminal activation function-1 (AF1) (A/B domain) that mediates transcriptional activation of target genes, the DNA-binding (C domain) involved in target promoter recognition, the flexible hinge domain (D domain) with a nuclear localization signal motif, and the C-terminal ligand-binding domain (LBD) (E/F domain), which contains the ligand binding pocket and is important for AF2 function, dimerization, and transcription activation. Notably, ERα and ERβ only share 59% similarity in their LBD, respectively, which allows their specific activation by distinct ligands [11]. The role of ER activation in various physiological processes is well documented; notably, the effects of estrogen deficiency in menopause or ovariectomized female subjects pointed out its participation in metabolism and energy homeostasis at central and peripheral levels [12,13,14].

Several studies have reported that estrogen receptor-beta (ERβ) is an attractive drug target for reducing body weight [12,13,14,15]. However, treatments with estradiol, the natural ligand of ERβ, also stimulate estrogen receptor alpha (ERα), causing several effects, such as hypertension, cerebrovascular events, myocardial infarction, and ovarian and endometrial cancer [16]. Therefore, studies focus on the development of selective ERβ activators. Isoquinolinone derivatives (β-LNGD1 and β-LNGD2) increase energy expenditure in brown adipose tissue (BAT) and inhibit white adipose tissue (WAT) lipogenesis in ovariectomy-induced obese mice by ERβ activation [12,14]. In another study, β-LNGD2 promoted the differentiation of mesenchymal stem cells into BAT instead of WAT, increasing mitochondriogenesis, mitochondrial function, and energy expenditure by ERβ activation [13]. Moreover, 4-(2-(3,5-dimethylisoxazol-4-yl)-1H-indol-3-yl)phenol, a selective ERβ activator, decreases visceral and subcutaneous WAT in the liver through the expression of genes that induce metabolic change, decreasing triglyceride synthesis by reducing de novo lipogenesis and increasing lipolysis in SAT and VAT leading to small size adipocytes [17]. Setchell et al. showed that S-equol (SE) has a better affinity for ERβ (Ki = 0.73 ± 0.2 nmol/L) than for ERα (Ki = 6.41 ± 1 nmol/L) [18], favoring weight loss in mice by reducing food intake [19]. Short SE exposure has also been reported to have a long-term inhibitory effect on adipogenesis in mouse 3T3-L1 cells [20]. Therefore, we carried out ligand-based virtual screening (LBVS), molecular docking (MD), and molecular dynamic simulations (MDS) to predict small molecules as new potential ERβ activators.

## 2. Results

### 2.1. Analysis of ERβ-Ligand Complexes

**Figure 1:**
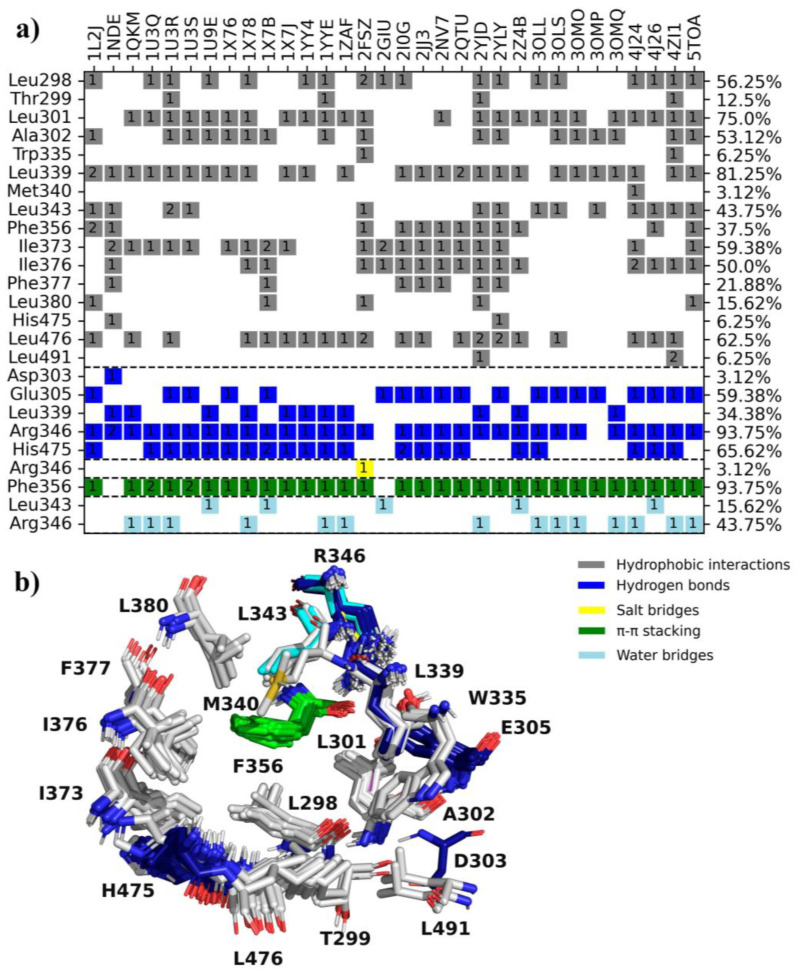
Analysis of interactions of the 32 co-crystallized ligands on the active site of ERβ. (**a**) ERβ-ligand interaction fingerprint; the right y-axis shows the percentage of participation of the residues in the interaction with the ligands, while the PDB codes are on the upper x-axis. The numbers in the cell indicate the number of times a residue interacts with the ligand. (**b**) Localization of the 19 aa residues that participate in ligand-ERβ interactions.

The 32 ERβ co-crystallized complexes in PDB were analyzed to identify amino acids (aa) and important interactions for ligand recognition. The results showed 25 relevant aa residues (Figure 1, y-axis) in the binding pocket on the active site of ERβ. Five main types of interactions were observed in ERβ, where hydrophobic interactions predominate, followed by hydrogen bonds, π-π stacking, water bridges, and salt bridges. The 25 aa residues on the active site of ERβ participating in ligand interactions are shown in Figure 1b.

### 2.2. MD Validation and Control Compounds Analysis

**Figure 2:**
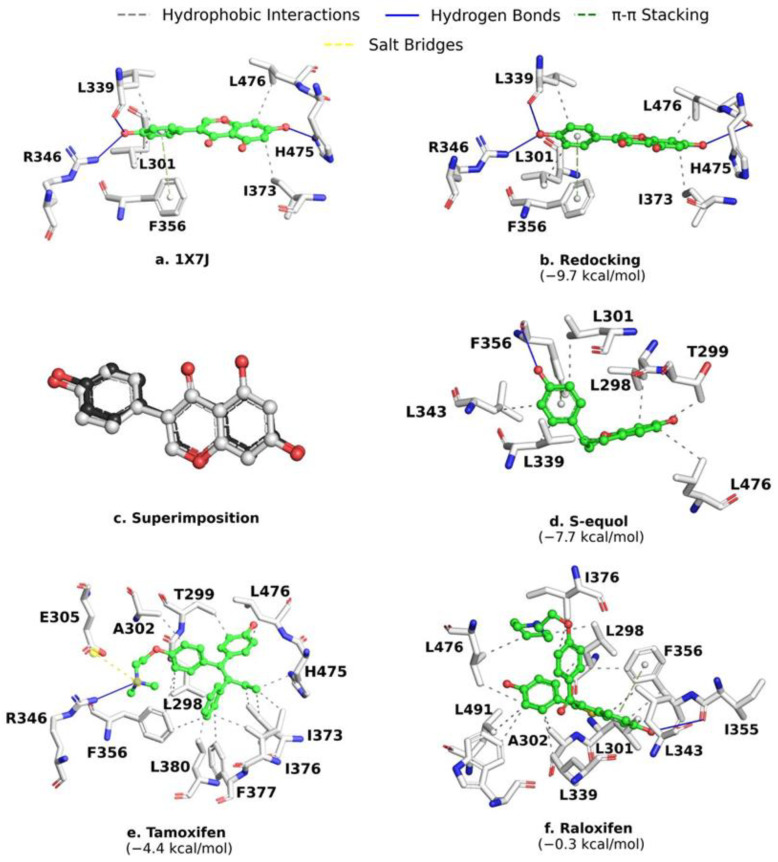
Re-docking of the co-crystallized ligand GEN on the active site of ERβ. (**a**) ERβ-GEN interactions at 1X7J; (**b**) ERβ-GEN interactions in the re-docking assay; (**c**) GEN structure superposition in docking (gray) and redocking (black); (**d**) SE; (**e**) tamoxifen; and (**f**) raloxifene on the site of ERβ. Values of ΔG_b_ are shown.

To validate the conditions of MD with AutoDock Vina 1.1, we first performed a re-docking assay between genistein (GEN) and ERβ. The obtained complex reproduced the same interactions with aa residues on the active site of ERβ as the original complex 1X7J. Notably, 8 out of the 25 most conserved aa residues are connected to GEN. Hydrophobic interactions predominated (L339, L301, L476, and I373), followed by hydrogen bonds (L339, R346, and H475), and π-π stacking (F356) (Figure 2a,b). The control compounds, GEN, SE, tamoxifen, and raloxifene (estrogen receptor modulators), showed ΔG_b_ values of −9.7 kcal/mol, −7.7 kcal/mol, −4.4 kcal/mol, and −0.3 kcal/mol, respectively (Figure 2).

The control compounds (SE, tamoxifen, and raloxifene) mainly had hydrophobic interactions with ERβ. Raloxifene and SE showed the hydrophobic interactions described with GEN (L339, L301, and L476), except with I373. They also presented the same π-π stacking interaction with F356, but hydrogen bonds were formed with different residues. Raloxifene interacts with I355, while SE binds F356. Tamoxifen formed the two hydrophobic interactions described with GEN (I373 and L476), a hydrogen bond with R346, and a salt bridge with E305. The π-π stacking interaction with F356 was also conserved (Figure 2).

### 2.3. LBVS by Structure Similarity

**Figure 3:**
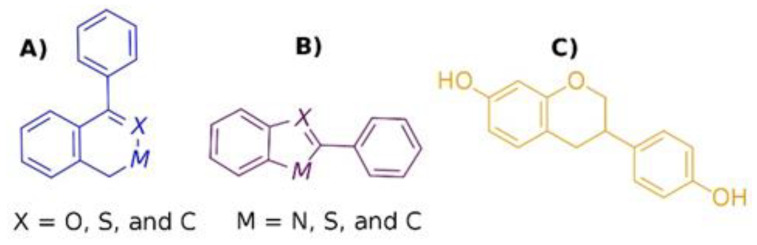
Scaffolds used to obtain new potential ERβ activators by LBVS in the ZINC15, PubChem, and MolPort databases. Scaffold (**A**,**B**) were obtained from co-crystalized ligands on ERβ, and scaffold (**C**) was obtained from S-equol (SE) structure.

The search for new ERβ activators in the ZINC15, PubChem, and MolPort databases using scaffolds A, B, and C (Figure 3) by structure similarity produced 2318, 24,571, and 3514 compounds, respectively. Of these compounds, 123, 1093, and 14 met Lipinski’s rule. Duplicate compounds were eliminated. A selection of molecules using a Tanimoto coefficient (TC) > 0.8, according to the scaffold, produced 29, 104, and 13 compounds (Table S2). Then, an MD analysis on the active site of ERβ using the cutoff point of ΔG_b_ = −4.4 kcal/mol (tamoxifen) led to the selection of 15, 58, and 11 compounds of scaffolds A, B, and C, respectively (Table S3). Finally, the interaction fingerprints of the compounds selected on the active site of ERβ were analyzed using the Open Drug Discovery Toolkit (ODDT). One compound of scaffold A, three compounds of scaffold B, and one compound of scaffold C were selected, applying a criterion of a TC > 0.7 with respect to the interaction fingerprint of the control compounds. However, these compounds are not commercially available; therefore, no further analysis was considered.

### 2.4. LBVS by Substructure Similarity

The search for new ERβ activators using scaffolds A, B, and C (Figure 3) by substructure led to 10,671, 46,315, and 6796 compounds, respectively. Among these, 203, 1097, and 100 met Lipinski’s rule. The duplicate compounds were eliminated; 189, 595, and 59 compounds from scaffolds A, B, and C remained (Table S2). Subsequently, 156 compounds from scaffold A, 625 compounds from scaffold B, and 90 compounds from scaffold C were obtained using the cutoff point of ΔG_b_ = −4.4 kcal/mol (Table S3). Afterward, 27, 72, and 15 compounds of scaffolds A, B, and C, respectively, were selected using the criterion of a TC > 0.7 with respect to the interaction fingerprint of the control compounds (Figures S2 and S3). Finally, the top ten (ΔG_b_ value) compounds from each scaffold were obtained. The ΔG_b_ values ranged from −9.2 to −10.3 kcal/mol for compounds of scaffold A (Figure S5), from −9.4 to −10.6 kcal/mol for compounds of scaffold B (Figure S6), and from −7.4 to −10.1 kcal/mol for compounds of scaffold C (Figure S7). Due to their commercial availability, only two compounds were acquired, a derivative of scaffold B, compound **2** (C2) (ΔG_b_ = −9.5 kcal/mol), and a derivative of SE, compound **1** (C1) (ΔG_b_ = −9.9 kcal/mol).

### 2.5. Drugs Repositioning

**Figure 4:**
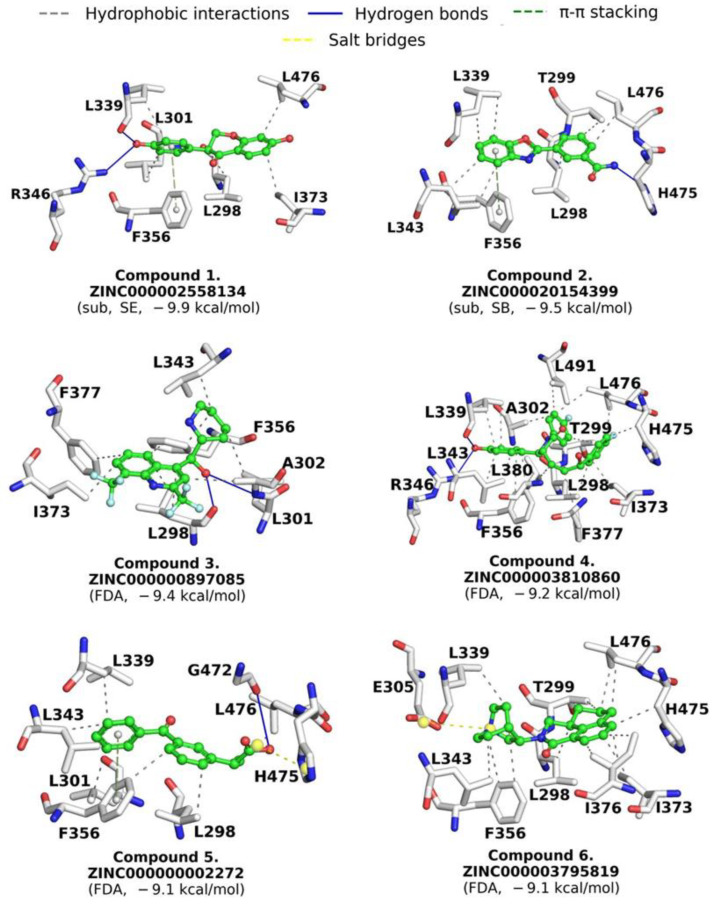
Interaction profile of potential ERβ activators. Compounds **1**–**6** are enlisted according to the ΔG_b_. Sub (substructure); SB (scaffold B); FDA (FDA-approved drugs).

A total of 1615 FDA-approved drugs were evaluated by MD on the active site of ERβ in a repositioning approach. After applying the previously used cutoff value of ΔG_b_ (−4.4 kcal/mol), 1103 compounds were obtained (Table S3). These were reduced to 149 using the criterion of a TC > 0.7 with respect to the interaction fingerprint of the control compounds (Figure S4). The top ten compounds had a ΔG_b_ ranging from −10.2 to −9.1 kcal/mol (Figure S8). Based on their commercial availability, four compounds were selected for further study: compound **3** (mefloquine, ΔG_b_ = −9.4 kcal/mol); compound **4** (ezetimibe, ΔG_b_ = −9.2 kcal/mol); compound **5** (ketoprofen, ΔG_b_ = −9.1 kcal/mol); and compound **6** (palonosetron, ΔG_b_ = −9.1 kcal/mol). These compounds have predominantly hydrophobic interactions (six to twelve), with six interactions conserved (L298, L339, L343, F356, I373, and L476). Compound **3** (C3) showed hydrogen bonds with L298 and A302, compound **4** (C4) with L339 and R346, and compound **5** (C5) with G472. Additionally, compound **5** established a salt bridge with H475 and compound **6** (C6) with E305; C1, C2 and C5 presented π-π stacking with F356 (Figure 4).

### 2.6. MDS Analysis

**Figure 5:**
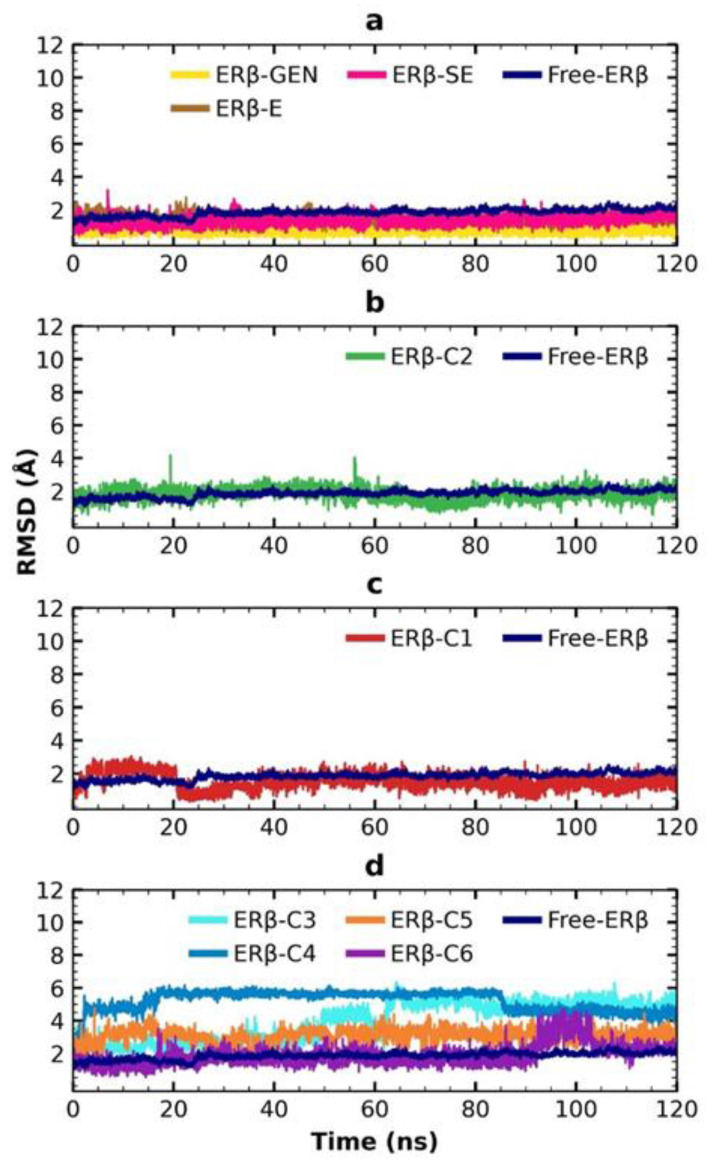
RMSD of the ERβ-activator complex simulation. (**a**) RMSD of the free ERβ and the control compounds; (**b**) compound derived from scaffold B; (**c**) SE derivative; and (**d**) FDA-approved drugs.

An MDS of 120 ns was performed with the GROMACS program to determine the stability and flexibility of the six selected compounds in complex with ERβ. First, RMSD values were calculated to describe the global movements throughout the 120 ns (Figure 5). The free ERβ shows an increase in RMSD values from 0.01 Å to 2.52 Å. The ERβ-GEN, ERβ-estradiol (ERβ-E), and ERβ-SE complexes showed changes in the RMSD values from 0.16 Å to 1.87 Å, from 0.63 Å to 2.77 Å, and from 0.44 Å to 3.2 Å, respectively. The fact that RMSD values remained higher in free ERβ compared with complexes suggests that the atomic positions of the ERβ protein are stabilized by interaction with the ligands. The fluctuations in each complex were 1.7 Å, 2.14 Å, and 2.76 Å, respectively, with ERβ-GEN being the most stable complex (ΔRMSD < 2 Å) (Figure 5a). Analysis of the RMSD box plot confirmed these observations (Figure S9a). A comparative analysis of RMSD fluctuations from ERβ-C1 to ERβ-C6 complexes indicated that the ERβ-C2 complex was the most stable with RMSD values from 0.3 Å to 4.17 Å (Figure 5b).

**Figure 6:**
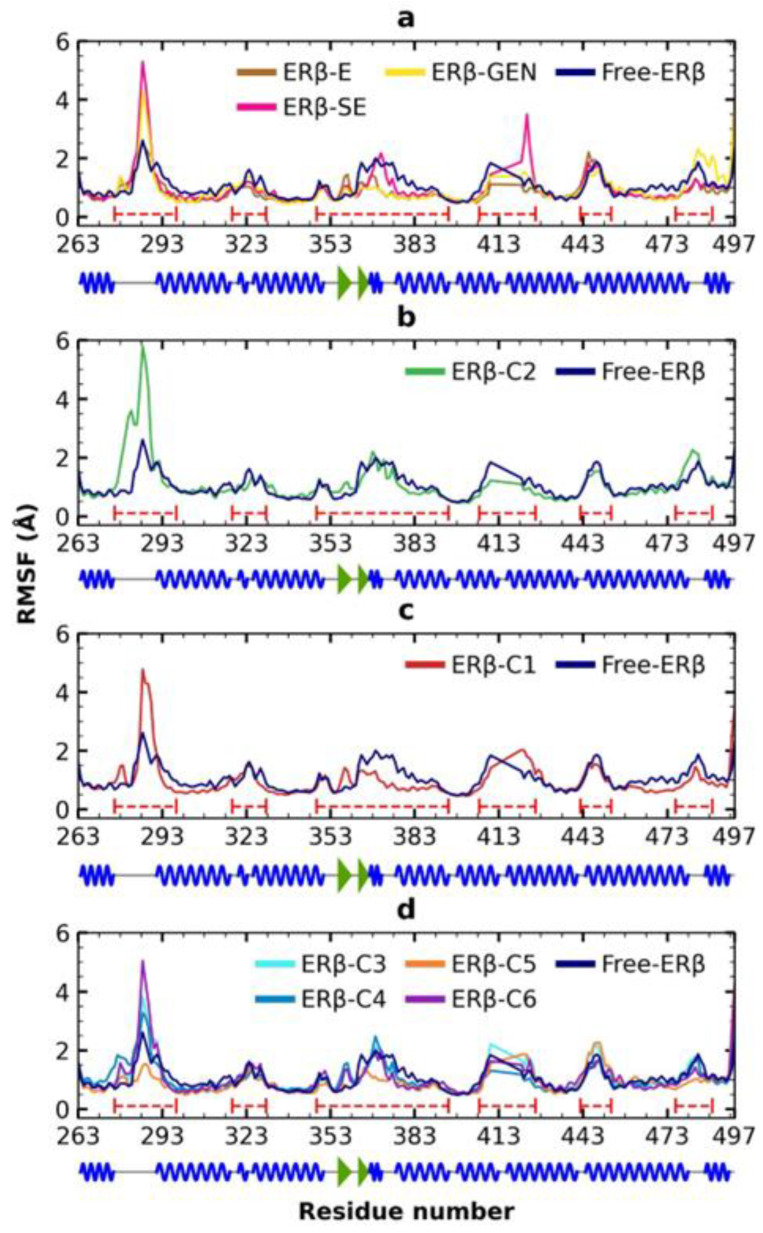
Influence of activator on the Cα of ERβ during the simulation. (**a**) RMSF of ERβ together with the control ligands. (**b**) RMSF of the scaffold derivative B. (**c**) RMSF of the SE derivative. (**d**) RMSF of FDA compounds. In 2D image of ERβ, the lines in gray represent loops; the spiral in blue are alpha helices, and the arrows in green are beta sheets. Dotted lines show regions with high fluctuations.

The RMSF values were analyzed to identify local movements of individual residues throughout the 120 ns of the MDS (Figure 6). Six fluctuation zones were identified in the free ERβ (Figure 6a, dotted lines). The region spanning residues 276–298 (region 1) had the highest mobility and corresponds to a loop; region 2 (318–330 aa) corresponds to loops and α-helices in which mobility can be attributed; region 3 (348–395 aa) corresponds to short segments of β-sheets and α-helices joined by loops that result in movements of the main chain; region 4 (410–421 aa) is an unresolved segment in the protein, which may explain the fluctuation; region 5 (442–453 aa) is a turn loop but is the continuation of the uncrystallized segment that could contribute to the fluctuation, and region 6 (476–489 aa) is the c-terminal formed by the loop—α-helix-loop.

**Figure 7:**
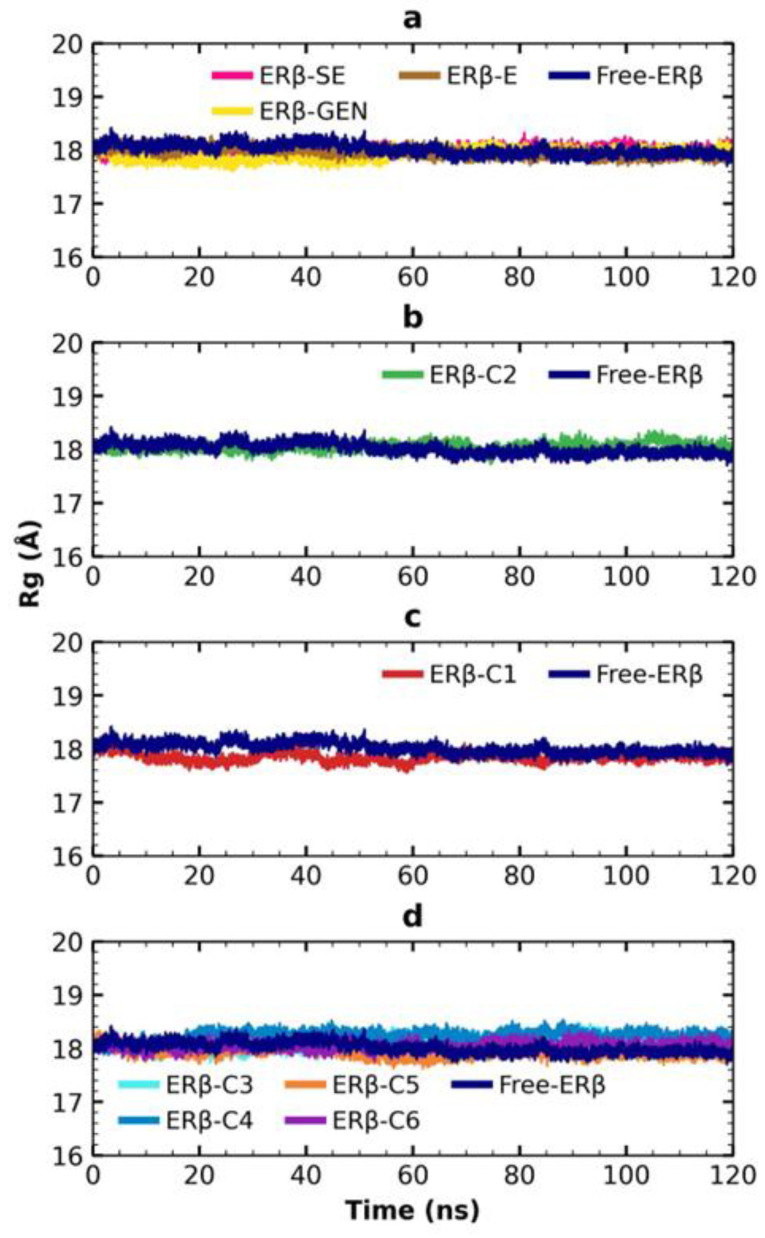
Rg of ERβ in the simulation. (**a**) control compounds; (**b**) derived from scaffold B; (**c**) derived from SE; and (**d**) FDA-approved drugs.

The radius of gyration (Rg) is another important parameter to measure the structural change in a protein during MDS [21]. The folding of free ERβ remains almost constant at around 18 Å during the 120 ns. The same observation occurs when ERβ interacts with the control compounds (Figure 7a). Congruently, the distribution of Rg values is in the same range in these four conditions (Figure S9i). The interaction of ERβ with molecules C1–C6 does not significantly influence the three-dimensional structure of ERβ since Rg also remains stable around 18 Å over the 120 ns of the MDS (Figure 7b–d). Globally, the dispersion of Rg is similar when ERβ is free or interacting with compounds C1 to C6 (Figure S9i–l).

The MMPBSA calculation is an efficient and reliable ΔG_b_ simulation method that characterizes molecular recognition in protein–ligand complexes in MDS. The complexes formed with the control compounds showed a ΔG_b_ of −27.01 to −32.17 kcal/mol, and the complex ERβ-C1-6 showed ΔG_b_ values of −22.65 to −36.5 kcal/mol (Table 1). In particular, the ERβ-C4 complex had a lower ΔG_b_ value (−36.5 kcal/mol) than the complexes formed with control compounds.

**Figure 8:**
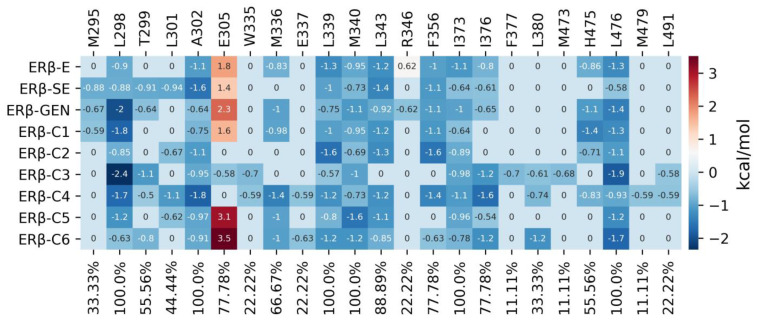
Heat map of the energy contribution of the residues in the ERβ. Only aa with a contribution of ΔG_b_ < −0.5 kcal/mol and ΔG_b_ > 0.5 kcal/mol were considered; the contribution values for each residue are in the box.

The decomposition of the residues that contribute to the binding energy was determined in the last 10 ns of the MDS with g_mmpbsa to understand which residues are the main contributors to the interaction between ERβ and different molecules. The results showed that 23 aa of ERβ bind to compounds C1–C6, where C4 interacts with the highest number of aa (18 aa) and has the best ΔG_b_. These 23 residues have ΔG_b_ values ranging from −2.4 to 3.5 kcal/mol (Figure 8).

### 2.7. ADMET Analysis

According to our experimental design, all selected molecules from the molecular docking are in accordance with the Lipinski rule, which predicts their drug-likeness. In addition, the assessment of their pharmacokinetics, including their physicochemical properties, medicinal chemistry properties, absorption, distribution, metabolism, excretion, and toxicity (ADMET), is a necessary step to confirm their relevance as drugs. Figure S10 shows the physicochemical and ADMET properties of the compounds C1–C6 previously selected.

First, the ADMET properties of compounds other than those of the FDA are covered. Compounds C1–C2 are predicted to have good solubility since they meet all the physicochemical characteristics and are promising drugs as they pass the drug-likeness rules for medicinal chemistry. In absorption, compounds C1–C2 show low bioavailability (F30%), while C2 is a substrate and inhibitor of P-glycoprotein (it expels xenobiotics). In distribution, both compounds have plasma protein binding (PPB) capabilities that reduce their bioavailability. This behavior is observed in unbound fractions (Fu) in plasma. C1 is a substrate for two CYP isoforms (CYP 2C9/2D6) and is an inhibitor of all CYP isoforms (CYP 1A2/2C19/2C9/2D6/3A4), indicating that these enzymes can be metabolized by these enzymes, while C2 is an inhibitor of a single CYP isoform. Regarding toxicity, C1 can be mutagenic (AMES toxicity) and cause rat acute oral toxicity and skin sensitization. C2 can cause drug-induced liver injury (DILI), generate a mitochondrial membrane potential (SR-MMP), and activate p53. Both C1 and C2 have adverse effects on the aryl hydrocarbon receptor (NR-AhR), the antioxidant response element (SR-ARE), and protein 5 containing the AAA domain of the ATPase family (SR-ATAD5). We do not consider those warnings for estrogen receptors (NR-ER and NR-ER-LBD).

FDA compounds (C3–C6) violate most of the ADMET parameters; however, they are the approved drugs with side effects reported to be related to toxicity. C3 is a substrate and a P-glycoprotein inhibitor. It is metabolized by CYP3A4 and CYP3A4 inhibitors. C4 is not a cytochrome P450 substrate or inhibitor; however, in silico prediction disagrees, showing that it is a substrate or an inhibitor. C5 is metabolized as the main enzyme by UDP-glucuronosyltransferase and secondarily by CYP2C enzymes. C6 is metabolized by CYP2D6, P450, CYP3A, and CYP1A2.

## 3. Discussion

ERβ has been considered a potential target for obesity treatment [12]; therefore, the search for new activators has been promoted. Among the 25 remaining aa residues, 16 established hydrophobic interactions with the ligands. Notably, L298, L301, A302, L339, I373, I376, and L476 were found in >50% of the 32 complexes, while three main residues (E305, R346, and H475) participate in hydrogen bonds in >50% of complexes. Interestingly, F356 forms a π-π stacking bond in 93% of the complexes. Less frequent interactions include salt bridges (Figure 1a). The 3D alignment of all ERβ-ligand complexes shows that the distribution of hydrophobic interactions is around the active site cavity, while hydrogen bonds are on opposite sides. The amino acid that favors the formation of π-π stacking is in the middle of all interactions (in 30 complexes of the 32 PDBs considered, Figure 1b).

In GEN redocking on the active site of ERβ, the RMSD was 0.30 Å. The ERβ-ligand complex co-crystallized had an RMSD of <2 Å [22]. These results validate the parameters used in our MD analysis. Tamoxifen and raloxifene, two FDA-approved drugs that modulate estrogen receptors [12,23,24], and SE, an ERβ activator [19], were included as reference compounds. SE had the best ΔG_b_ value. This result agrees with the low Ki value of 0.73 ± 0.2 nmol/L and the high experimental affinity of SE for ERβ [18]. Interestingly, this ΔG_b_ value is near the ΔG_b_ value described for coumestrol (−8.5 kcal/mol), another phytoestrogen and ERβ activator that establishes hydrogen bonding interactions with G305, L339, R346, G472, and H475 [25].

The search for compounds by similarity and substructure in ZINC15, PubChem, and MolPort and the application of a series of criteria to filter these compounds led to the selection of four compounds by similarity; however, these compounds were discarded due to their lack of availability. In contrast, of the compounds chosen by substructure, two were selected, C1 and C2, which established five hydrophobic interactions with ERβ. Both interact with L298, L339, and L476. Additionally, C1 shows interactions with L301 and I373, and C2 with T299 and L343. The hydrogen bonds are maintained with L339 and R346 (C1) and H475 (C2). Both compounds interact with F356 through π-π stacking (Figure 3).

The FDA drug repositioning approach allowed four compounds to be selected. Mefloquine (C3) is a drug used to prevent or combat malaria; however, in a neurology study, it caused weight loss in patients, although the cause was not explored. Additionally, mefloquine can cause neuropsychiatric side effects and abnormal heart rhythms [26]. These effects could dismiss its potential use in obesity.

Ezetimibe (C4) is a drug that reduces cholesterol and has serious uncommon side effects. This drug caused a reduction in abdominal visceral fat in a study of patients with metabolic syndrome [27]. In another study, ezetimibe improved body weight [28]. Cho et al. [29] analyzed the effect of ezetimibe on adipose tissue. Their results showed a reduction in the size of adipocytes, suggesting that ezetimibe affects pyruvate dehydrogenase kinase 2, an enzyme that regulates the metabolism of glucose and fatty acid metabolism. These previous results suggest that ezetimibe could be a potential drug for the pharmacological treatment of obesity; however, more studies are necessary to confirm its effect on ERβ or other targets.

Ketoprofen (C5) is a nonsteroidal anti-inflammatory drug with analgesic and antipyretic effects that, administrated to mice with a high-fat diet, causes a reduction in body weight [30]. Therefore, ketoprofen could be another potential drug for combating obesity; however, it is also necessary to confirm its mechanism of action. Finally, palonosetron (C6) is a drug used to prevent nausea and vomiting, with no reports of weight problems [31]. Although the central serotonergic system reduces food intake and lower body weight, palonosetron, a serotonin antagonist, could have a null effect on body weight. However, its biological effect on ERβ must be confirmed.

One way to improve the prediction of MD is to take the predicted pose of the compound and use an MDS to determine the compound’s stability over time. When evaluating the six selected compounds, it was shown that the ERβ-C2 complex had the best RMSD. This result was corroborated by the RMSD box plot between ~0.8 Å and ~2.8 Å (Figure S9b). The ERβ-C1 complex presents an increase in RMSD in the first 20 ns of the MDS, from 0.48 Å to 3.05 Å; then, the RMSD values decrease to 0.29 Å and progressively increase to reach 2.74 Å at 120 ns. Interestingly, the RMSD values of ERβ-C1 and ERβ-C2 remain lower than those of free ERβ at the end of the MDS, suggesting that ligand binding stabilizes the protein structure. The ERβ-C3, ERβ-C4, ERβ-C5, and ERβ-C6 complexes (FDA-approved drugs) showed the highest variations in RMSD with values ranging from 0.48–6.37 Å, 0.47–6.17 Å, 0.42–4.76 Å, and 0.55–4.72 Å, respectively. Among these, the ERβ-C6 complex is the most stable, with an RMSD value of less than 3 Å. Congruently, the distribution of RMSD values of free ERβ and ERβ-C6 is almost similar, while it moves to higher ranges in the case of ERβ-C3, ERβ-C4, and ERβ-C5 (Figure S9d).

The analysis of RMSF values highlights high mobility in the loops of ERβ. This result agrees with previous work by Zafar et al. [25], who reported five oscillation zones in ERβ: ~280–~300 aa, ~310–~330 aa, ~340–~380 aa, ~410–~420 aa, and ~480–~500 aa. Variations in RMSF elements of the secondary structure of ERβ in complex with the control compounds during the simulation occur in the same six mobile regions previously described in free ERβ; however, movements are generally of greater magnitude (Figure 5a). The same fluctuations are observed when ERβ forms complexes with compounds C1–C6 (Figure 5b–d). The distribution of RMSF values does not significantly change when ERβ is free or in complex with control ligands or compounds C1 to C6 (Figure S9e–h).

The integrity of the three-dimensional structure of ERβ with the Rg was confirmed. The dispersion range was <0.73 Å and with an average of 17.90 Å < Rg < 18.30 Å (Figure S9i–l) for all simulations, which is similar to the ~18.2 Å value obtained by Zafar et al. [25] and consistent with the Rg of 18.0 ± 0.1 Å described for α/β proteins of 201–250 aa [32]. These data indicate that ERβ conformation remains compact during the simulation; therefore, the little instability observed for C3, C4 and C5 could not be due to modifications of the protein conformation.

The calculation of the ΔG_b_ with MMPBSA shows that ERβ-C4 is the complex with the best ΔG_b_. On the other hand, the stable complex ERβ-C2 has a ΔG_b_ of −23.33 ± 0.3 kcal/mol; when analyzing the residues that participate in the binding of compounds in the MDS, notably, the relevant aa identified in >50% of the complexes include T299, H475, M336, F356, I376, L343, L298, A302, L339, M340, I373, and L476, which favorably contribute to interactions (having negative ΔG_b_ values), and E305, which promotes the interaction of C3 but not of other molecules, and R346 that does not help to estradiol binding (Figure 7). The detection of these 13 aa in most simulations, including the complexes formed with the six new molecules, suggests that they can be crucial for ligand binding.

ADMET analysis of non-FDA compounds shows that both compounds have good solubility, bioavailability, and absorption, where C1 appears to be slightly toxic; however, both compounds are promising molecules for ERβ activation, and for FDA drugs, they do not meet some ADMET parameters; however, these side effects are documented. Therefore, ADMET in silico prediction agrees with that reported in the literature.

## 4. Materials and Methods

### 4.1. Analysis of ERβ and Its Ligands

The 32 structures of human ERβ co-crystalized with a ligand (Figure S1) were selected from the Protein Data Bank (PDB) and analyzed with the Protein–Ligand Interaction Profiler (PLIP) program [33] to identify non-covalent interactions and establish the common interaction profile. The result was used as a criterion in the selection of new potential ERβ activators.

### 4.2. Preparation of ERβ Tridimensional Structure

The PDB file 1X7J with high resolution (2.30 Å), only a gap, and no mutations corresponding to the crystal structure of ERβ (Ligand Binding Domain, LBD) in complex with genistein (GEN) were selected for molecular docking (MD) analysis. Different molecules interacting with ERβ were removed from the protein with the Chimera program, polar hydrogens were added with DockPrep, and the side chains were repaired [34]. Finally, the ERβ file was converted to the PDBQT format by adding Gasteiger charges using MGTools 1.5.6 [35].

### 4.3. MD Validation and Analysis of Control Compounds

Initially, an MD analysis of GEN was performed on the active site of ERβ with AutoDock Vina 1.1. The center of the box for MD was X = 29.854 Å, Y = 36.297 Å, and Z = 38.964 Å, with a box size of 20 Å in XYZ. SE and two approved FDA drugs as estrogen receptor modulators (raloxifene and tamoxifen) were used as control compounds in the MD assays. The free energy of binding (ΔG_b_ = −4.4 kcal/mol) of tamoxifen, a competitive antagonist of estradiol, the natural ERβ ligand, was used as a cut-off value to select potential ERβ activators.

### 4.4. Identification of New Potential ERβ Ligands

#### 4.4.1. LBVS

Two strategies were considered for the LBVS: (1) selection of two scaffolds (Figure 3A,B) from the common structures of the 32 ligands co-crystallized with the human Erβ; and (2) selection of SE structure (Figure 3C) as a scaffold [18,19]. LBVS was performed in the ZINC15, PubChem, and MolPort databases using the structure similarity [Tanimoto coefficient (TC) > 0.8] and substructure. Subsequently, the Lipinski rule was used as a criterion of selection. Duplicate molecules were eliminated. Finally, the minimization of compounds and addition of polar hydrogens were performed with Open Babel [36].

#### 4.4.2. Drug Repositioning

In another strategy, the FDA-approved drugs included in the ZINC15 database were analyzed through MD on the active site of ERβ. All FDA drugs were converted into the PDBQT format for MD assays. MD assays between ERβ and FDA drugs were performed using AutoDock Vina 1.1 [35].

#### 4.4.3. Interaction Fingerprint Analysis

The selected compounds by LBSV were analyzed with the simple interaction fingerprint methodology using Open Drug Discovery Toolkit (ODDT) software [37] to identify molecules with an interaction fingerprint similar to the control compounds using a TC > 0.7. Then, the compounds were ordered according to the ΔG_b_ value from each scaffold used (A, B, and C) and FDA-approved drugs. Of the top ten, the six compounds (C1–C6) with commercial availability were considered for further computational analysis.

#### 4.4.4. Molecular Dynamics Simulations (MDS)

The stability of the selected and control compounds (GEN, estradiol, and SE) in complex with ERβ was analyzed by MDS. The best pose of each compound was recovered from the ERβ-ligand complex from the MD analysis. The topology of the ligand poses was generated with the AnteChamber Python Parser interface (ACPYPE) [38,39]. On the other hand, the topology of the ERβ was obtained with GROMACS software version 2018.4 [40]. All molecular simulations were run on GROMACS with the AMBER force field. The complex was solvated in a dodecahedron box with the TIP3P water model at ten angstroms from the walls; the system was neutralized with Na^+^ and Cl^−^ ions followed by 50,000-fold energy minimization, and the system was equilibrated at 100 ps under the NVT (number of particles, volume, and temperature) and NPT (number of particles, pressure, and temperature). Finally, the simulation was carried out at 300 K with an atmosphere of **1** for 120 ns [41,42].

#### 4.4.5. MDS Trajectories Analysis

The atomic characteristics of the different complexes were compared using the analysis tools included in the GROMACS software. Particularly, values of root mean square deviation (RMSD) between α-carbons and ligand, root mean square fluctuation (RMSF) of α-carbons together with the structure in 2D of the Erβ, and the radius of gyration (Rg), were used to determine complex stability. Values of ΔG_b_ were obtained in the 50 snapshots of the last 10 ns of the MDS trajectory by calculating the Molecular Mechanics Poisson–Boltzmann Surface Area (MMPBSA) of each complex with the g_mmpbsa program [43]; the residues involved in the interaction and their energy contribution were obtained with the MmPbSaDecomp.py script.

#### 4.4.6. Prediction of ADMET Properties

The prediction of absorption, distribution, metabolism, excretion, and toxicity (ADMET) properties for the selected molecules (C1–C6) was carried out by entering the code smiles in the ADMETlab 2.0 web server [44], using the available parameters: physicochemical properties; medicinal chemistry properties; absorption; distribution; metabolism; excretion; and toxicity.

## 5. Conclusions

LBVS with scaffolds A, B, and C allowed identifying two new compounds from the substructure search for four compounds (mefloquine, ezetimibe, ketoprofen, and palonosetron) from the FDA repositioning; these are potential ERβ activators. Previous research confirms that ezetimibe and ketoprofen have anti-obesity effects. In addition, the MDS indicates that C1, C2 and C6 form a stable interaction on the active site of ERβ. These results suggest that they could have good in vitro activity. Additionally, these compounds could be considered in developing new anti-obesity agents.

## Figures and Tables

**Table 1:** ΔG_b_ (kcal/mol) of the complexes in the MDS calculated with MMPBSA.

Complex	ΔE_vdw_	ΔE_ele_	ΔG_polar_	ΔG_SA_	ΔG_b_
ERβ-E	−41.24 ± 0.30	−8.15 ± 0.52	21.09 ± 0.25	−3.86 ± 0.02	−32.17 ± 0.37
ERβ-SE	−35.77 ± 0.43	−14.68 ± 0.26	26.87 ± 0.27	−3.41 ± 0.02	−27.01 ± 0.41
ERβ-GEN	−37.72 ± 0.35	−14.43 ± 0.22	28.65 ± 0.18	−3.49 ± 0.02	−27.01 ± 0.30
ERβ-C1	−34.08 ± 0.38	−13.69 ± 0.18	27.02 ± 0.19	−3.52 ± 0.02	−24.27 ± 0.34
ERβ-C2	−37.72 ± 0.25	−4.33 ± 0.14	22.1 ± 0.19	−3.38 ± 0.02	−23.33 ± 0.30
ERβ-C3	−44.71 ± 0.30	−1.87 ± 0.18	17.94 ± 0.16	−4.47 ± 0.02	−33.11 ± 0.30
ERβ-C4	−56.91 ± 0.28	−3.54 ± 0.4	28.89 ± 0.41	−4.92 ± 0.02	−36.5 ± 0.34
ERβ-C5	−38.96 ± 0.29	−4.4 ± 0.14	24.4 ± 0.30	−3.7 ± 0.02	−22.65 ± 0.36
ERβ-C6	−43.79 ± 0.27	−1.61 ± 0.11	20.07 ± 0.44	−4.17 ± 0.02	−29.55 ± 0.51

ΔE_vdw_, van der Waal energy; ΔE_ele_, electrostatic energy; ΔG_polar_, polar solvation energy; ΔG_SA_, SASA energy.

## Data Availability

Not applicable.

## Supplementary Materials

The following supporting information can be downloaded at: https://www.mdpi.com/article/10.3390/molecules28114389/s1. Table S1. PDB ID of estrogen receptor beta used in this study; Table S2. Number of compounds for each stage of virtual screening; Table S3. Post-selection of potential ERβ activators by groups; Figure S1. Co-crystallized ligands of estrogen receptor beta (ERβ). Each compound is identified by its PDB code. Compounds highlighted in blue were used to generate scaffold A and those used for scaffold B are in purple; Figure S2. Interaction fingerprint of the selected compounds derived from the scaffolds A and B with a tanimoto coefficient > 0.7. Name of the compounds in the graph; Figure S3. Interaction fingerprint of s-equol analogs with respect to control compounds. Name of the compounds in the graph; Figure S4. Selection of FDA compounds with interaction fingerprint similar to control compounds. Name of the compounds in the graph; Figure S5. Interaction profile of the top ten compounds derived from scaffold A, docked to the active site of ERβ. The number at the end of each type of interaction is the total number of interactions for each compound; Figure S6. Interactions of the top ten scaffold B compounds docked at the active site of the ERβ; Figure S7. Interactions of the top ten compounds of s-equol analogs on the active site of ERβ; Figure S8. The top ten FDA compounds docked to the ERβ active site; Figure S9. Box plot of RMSD (a-d), RMSF (e-h) and Rg (i-l). The graphs are ordered according to the median, the average is represented by an “x”, the points denote outliers; Figure S10. Evaluation ADMET of compounds C1-6.

## Sample Availability

Samples of the compounds are available from the authors.
