# Supplementary material for: Ligand-Based Virtual Screening, Molecular Docking, and Molecular Dynamic Simulations of New β-Estrogen Receptor Activators with Potential for Pharmacological Obesity Treatment
Source: Molecules. 2023 May 27;28(11):4389. doi: 10.3390/molecules28114389 (PMC10254148; doi:10.3390/molecules28114389)
Supplement: Supplementary file 1 [file molecules-28-04389-s001.zip › molecules-2414741-supplementary.pdf]

# Ligand-based virtual screening, molecular docking, and molecular dynamic simulations of new β-estrogen receptor activators with potential for pharmacological obesity treatment

Domingo Mendez-Alvarez[1], María F. Torres-Rojas[2], Edgar E. Lara-Ramírez[1], Laurence A. Marchat[2], Gildardo Rivera[1*]

[1] Laboratorio de Biotecnología Farmacéutica, Centro de Biotecnología Genómica, Instituto Politécnico Nacional, 88710 Reynosa, México.

[2] Laboratorio de Biomedicina Molecular 2, Escuela Nacional de Medicina y Homeopatía, Instituto Poli-técnico Nacional, Ciudad de México, México.

**Supplementary material**

**Table S1**. PDB ID of estrogen receptor beta used in this study.

| PDB ID | Descripcion | PDB ID | Descripcion |
|---|---|---|---|
| 1L2J | Estrogen receptor beta, 271 aa | 2I0G | Estrogen receptor beta, 257 aa |
| 1NDE | Estrogen receptor beta, 255 aa | 2JJ3 | Estrogen receptor beta, 257 aa |
| 1QKM | Estrogen receptor beta, 255 aa | 2NV7 | Estrogen receptor beta, 238 aa |
| 1U3Q | Estrogen receptor beta, 240 aa | 2QTU | Estrogen receptor beta, 257 aa |
| 1U3R | Estrogen receptor beta, 241 aa | 2YJD | Estrogen receptor beta, 240 aa |
| 1U3S | Estrogen receptor beta, 240 aa | 2YLY | Estrogen receptor beta, 240 aa |
| 1U9E | Estrogen receptor beta, 241 aa | 2Z4B | Estrogen receptor beta, 257 aa |
| 1X76 | Estrogen receptor beta, 240 aa | 3OLL | Estrogen receptor beta, 240 aa |
| 1X78 | Estrogen receptor beta, 240 aa | 3OLS | Estrogen receptor beta, 240 aa |
| 1X7B | Estrogen receptor beta, 240 aa | 3OMO | Estrogen receptor beta, 240 aa |
| 1X7J | Estrogen receptor beta, 240 aa | 3OMP | Estrogen receptor beta, 240 aa |
| 1YY4 | Estrogen receptor beta, 268 aa | 3OMQ | Estrogen receptor beta, 240 aa |
| 1YYE | Estrogen receptor beta, 268 aa | 4J24 | Estrogen receptor beta, 240 aa |
| 1ZAF | Estrogen receptor beta, 238 aa | 4J26 | Estrogen receptor beta, 240 aa |
| 2FSZ | Estrogen receptor beta, 246 aa | 4ZI1 | Estrogen receptor beta, 249 aa |
| 2GIU | Estrogen receptor beta, 241 aa | 5TOA | Estrogen receptor beta, 249 aa |

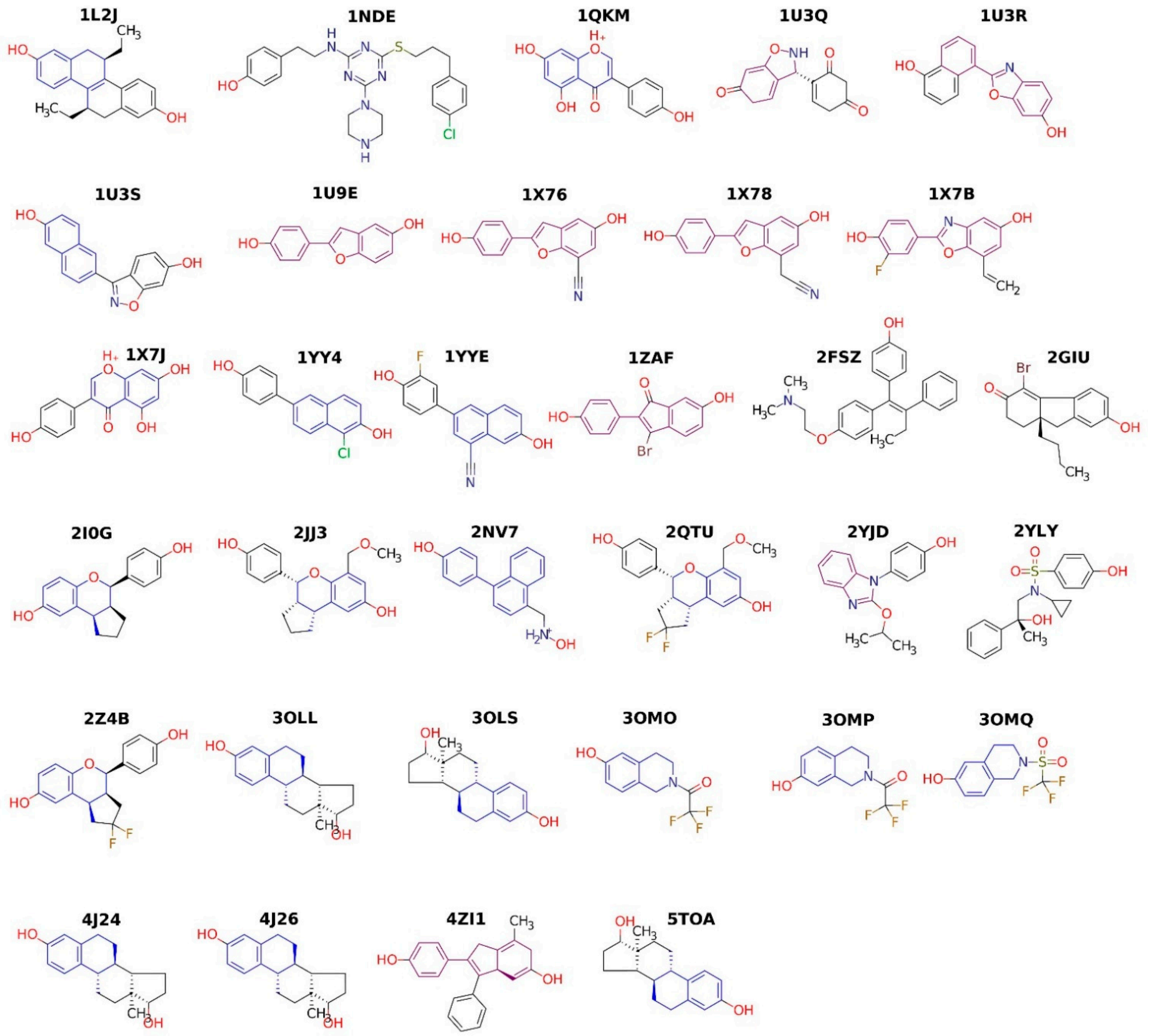


**Figure S1.** Co-crystallized ligands of estrogen receptor beta (ERβ). Each compound is identified by its PDB code. Compounds highlighted in blue were used to generate scaffold A and those used for scaffold B are in purple.

**Table S2.** Number of compounds for each stage of virtual screening.

| | Sim | | | Sub | | | |
|---|---|---|---|---|---|---|---|
| **Criteria** | **SA** | **SB** | **SE** | **SA** | **SB** | **SE** | **Total** |
| **Compounds retrieved from the database** | 2318 | 24571 | 3514 | 10671 | 46315 | 6796 | 94185 |
| **Lipinski's rule** | 123 | 1093 | 14 | 203 | 1097 | 100 | 2630 |
| **Elimination of duplicate compounds** | 109 | 768 | 13 | 189 | 595 | 59 | 1733 |
| **Compounds with a similarity > 0.8** | 29 | 104 | 13 | 189 | 595 | 59 | 989 |

Sim, similarity; Sub, substructure; SA, Scaffold A; SB, Scaffold B; SE, S-equol.

**Table S3.** Post-selection of potential ERβ activators by groups.

| | | Sim | | | Sub | | | |
|---|---|---|---|---|---|---|---|---|
| **Criteria** | **FDA** | **SA** | **SB** | **SE** | **SA** | **SB** | **SE** | **Total** |
| **Cut-off value (-4.4 kcal/mol)** | 1103 | 15 | 58 | 11 | 156 | 625 | 90 | 2058 |
| **Interaction fingerprint** | 149 | 1 | 3 | 1 | 27 | 72 | 15 | 268 |

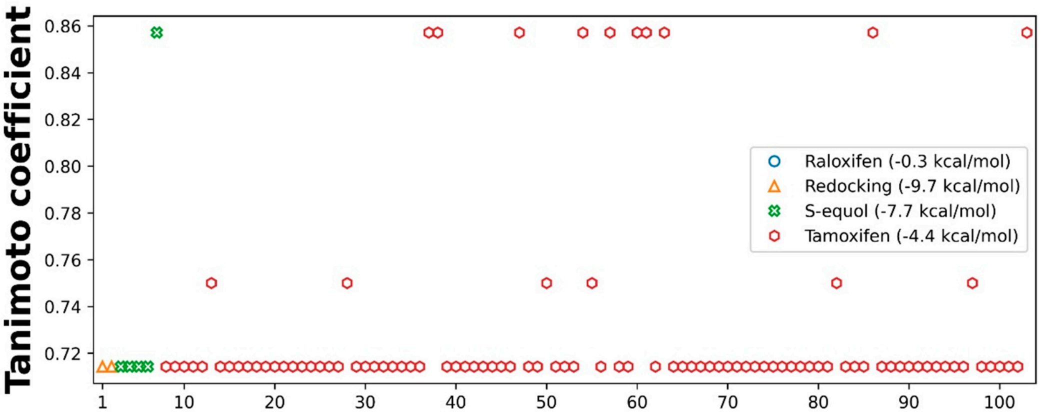


**Figure S2.** Interaction fingerprint of the selected compounds derived from the scaffolds A and B with a tanimoto coefficient > 0.7. Name of the compounds in the graph: 1: ZINC000000235493_sub_S2.4 (-9.1 kcal/mol); 2: ZINC000000558601_sub_S2.4 (-8.3 kcal/mol); 3: pubchem16096242_sub_S2.7 (-8.5 kcal/mol); 4: pubchem16114609_sub_S2.9 (-8.7 kcal/mol); 5: pubchem44550346_sub_S2.7 (-8.2 kcal/mol); 6: ZINC000006720868_sub_S1.3 (-7.4 kcal/mol); 7: ZINC000043152477_sub_S2.6 (-6.7 kcal/mol); 8: pubchem102029570_sub_S2.7 (-7.0 kcal/mol); 9: pubchem102159833_sub_S2.7 (-8.8 kcal/mol); 10: pubchem11290215_sub_S2.6 (-10.6 kcal/mol); 11: pubchem134968537_sub_S2.9 (-9.1 kcal/mol); 12: pubchem13901656_sub_S2.6 (-10.0 kcal/mol); 13: pubchem14387309_sub_S2.6 (-10.3 kcal/mol); 14: pubchem14387311_sub_S2.6 (-9.8 kcal/mol); 15: pubchem147054884_sub_S2.9 (-9.1 kcal/mol); 16: pubchem14920722_sub_S2.7 (-7.9 kcal/mol); 17: pubchem21032991_sub_S2.7 (-7.3 kcal/mol); 18: pubchem3027594_sim_S2.4 (-9.5 kcal/mol); 19: pubchem3784895_sub_S2.7 (-9.3 kcal/mol); 20: pubchem4253040_sub_S2.4 (-7.8 kcal/mol); 21: pubchem50850272_sub_S2.7 (-8.8 kcal/mol); 22: pubchem53423166_sub_S2.7 (-8.9 kcal/mol); 23: pubchem53497102_sim_S2.4 (-8.6 kcal/mol); 24: pubchem87145216_sub_S2.9 (-8.8 kcal/mol); 25: pubchem90477262_sub_S2.7 (-8.4 kcal/mol); 26: pubchem92045574_sub_S2.7 (-8.7 kcal/mol); 27: pubchem95910620_sub_S2.7 (-8.4 kcal/mol); 28: ZINC000000006701_sub_S2.7 (-9.0 kcal/mol); 29: ZINC000000019637_sub_S2.7 (-8.6 kcal/mol); 30: ZINC000000079780_sub_S1.3 (-8.2 kcal/mol); 31: ZINC000000099912_sub_S1.10 (-7.8 kcal/mol); 32: ZINC000000099914_sub_S1.10 (-5.5 kcal/mol); 33: ZINC000000107820_sub_S1.3 (-9.5 kcal/mol); 34: ZINC000000207155_sub_S1.10 (-6.9 kcal/mol); 35: ZINC000000207161_sub_S1.10 (-4.8 kcal/mol); 36: ZINC000000235985_sub_S2.7 (-8.5 kcal/mol); 37: ZINC000000273801_sub_S1.3 (-8.1 kcal/mol); 38: ZINC000000329772_sub_S1.3 (-8.3 kcal/mol); 39: ZINC000000349576_sub_S2.3 (-8.8 kcal/mol); 40: ZINC000000392849_sub_S1.12 (-10.0 kcal/mol); 41: ZINC000000506694_sub_S2.7 (-8.7 kcal/mol); 42: ZINC000001433321_sub_S2.7 (-8.3 kcal/mol); 43: ZINC000001650698_sub_S1.3 (-7.8 kcal/mol); 44: ZINC000001665251_sub_S1.12 (-9.2 kcal/mol); 45: ZINC000001669547_sub_S1.12 (-9.5 kcal/mol); 46: ZINC000001736836_sub_S1.3 (-5.6 kcal/mol); 47: ZINC000002584535_sub_S2.7 (-8.8 kcal/mol); 48: ZINC000002584539_sub_S2.7 (-8.5 kcal/mol); 49: ZINC000002997427_sub_S1.3 (-7.4 kcal/mol); 50: ZINC000003109197_sub_S1.3 (-7.7 kcal/mol); 51: ZINC000003844826_sub_S2.3 (-9.3 kcal/mol); 52: ZINC000003844883_sub_S2.3 (-9.0 kcal/mol); 53: ZINC000004571345_sub_S1.3 (-7.8 kcal/mol); 54: ZINC000004713938_sub_S2.3 (-9.4 kcal/mol); 55: ZINC000004736776_sub_S2.4 (-7.7 kcal/mol); 56: ZINC000005361254_sub_S2.9 (-7.4 kcal/mol); 57: ZINC000013118874_sub_S2.3 (-9.3 kcal/mol); 58: ZINC000016162088_sub_S2.7 (-7.8 kcal/mol); 59: ZINC000018051528_sub_S2.7 (-9.1 kcal/mol); 60: ZINC000019909740_sub_S1.12 (-9.5 kcal/mol); 61: ZINC000019909743_sub_S1.12 (-9.3 kcal/mol); 62: ZINC000025495200_sub_S2.5 (-7.3 kcal/mol); 63: ZINC000032264139_sub_S2.7 (-9.3 kcal/mol); 64: ZINC000032271185_sub_S1.6 (-9.6 kcal/mol); 65: ZINC000034283373_sub_S2.7 (-9.2 kcal/mol); 66: ZINC000035075930_sub_S2.7 (-8.4 kcal/mol); 67: ZINC000035963070_sim_S1.10 (-8.2 kcal/mol); 68: ZINC000036406739_sub_S1.12 (-9.1 kcal/mol); 69: ZINC000038841808_sub_S1.12 (-9.0 kcal/mol); 70: ZINC000038885998_sub_S2.5 (-7.8 kcal/mol); 71: ZINC000039120152_sim_S2.6 (-8.6 kcal/mol); 72: ZINC000039189287_sub_S2.5 (-8.5 kcal/mol); 73: ZINC000039189289_sub_S2.5 (-8.6 kcal/mol); 74: ZINC000039212519_sub_S2.7 (-9.0 kcal/mol); 75: ZINC000039304508_sub_S1.3 (-8.9 kcal/mol); 76: ZINC000045919981_sub_S2.7 (-8.9 kcal/mol); 77: ZINC000045923438_sub_S2.7 (-8.9 kcal/mol); 78: ZINC000045946712_sub_S2.7 (-9.5 kcal/mol); 79: ZINC000045946717_sub_S2.7 (-9.3 kcal/mol); 80: ZINC000045946736_sub_S2.7 (-9.4 kcal/mol); 81: ZINC000071769464_sub_S2.9 (-9.4 kcal/mol); 82: ZINC000071780618_sub_S2.6 (-7.8 kcal/mol); 83: ZINC000071784641_sub_S2.3 (-10.3 kcal/mol); 84: ZINC000096027552_sub_S1.3 (-7.8 kcal/mol); 85: ZINC000100365916_sub_S2.3 (-8.8 kcal/mol); 86: ZINC000100496590_sub_S2.6 (-9.0 kcal/mol); 87: ZINC000100531228_sub_S2.3 (-6.8 kcal/mol); 88: ZINC000103184740_sub_S2.12 (-8.7 kcal/mol); 89: ZINC000103184747_sub_S2.12 (-8.5 kcal/mol); 90: ZINC000103184834_sub_S2.12 (-9.4 kcal/mol); 91: ZINC000103185949_sub_S2.12 (-8.7 kcal/mol); 92: ZINC000103187202_sub_S2.12 (-9.4 kcal/mol); 93: ZINC000147535771_sub_S1.3 (-9.4 kcal/mol); 94: ZINC000169314377_sub_S1.3 (-9.5 kcal/mol); 95: ZINC000196467451_sub_S2.5 (-7.2 kcal/mol); 96: ZINC000199434926_sub_S2.3 (-8.6 kcal/mol); 97: ZINC000254797150_sub_S2.3 (-8.6 kcal/mol); 98: ZINC000584596711_sub_S2.9 (-8.7 kcal/mol); 99: ZINC000584596772_sub_S2.9 (-9.4 kcal/mol); 100: ZINC000584596773_sub_S2.9 (-10.1 kcal/mol); 101: ZINC000584649928_sub_S2.9 (-9.0 kcal/mol); 102: ZINC000020154399_sub_S2.4 (-9.5 kcal/mol); 103: ZINC001088689404_sub_S1.12 (-10.3 kcal/mol).

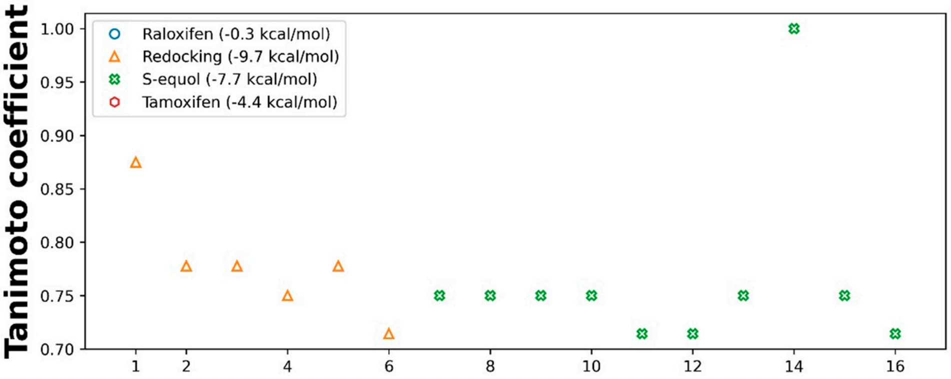


**Figure S3.** Interaction fingerprint of s-equol analogs with respect to control compounds. Name of the compounds in the graph. 1: ZINC000000388661_sub (-9.7 kcal/mol); 2: ZINC000000899786_sub (-10.1 kcal/mol); 3: ZINC000002558134_sub (-9.9 kcal/mol); 4: ZINC000003978471_sub (-8.7 kcal/mol); 5: ZINC000014811582_sub (-9.8 kcal/mol); 6: ZINC000014820588_sub (-8.8 kcal/mol); 7: ZINC000000899787_sub (-7.6 kcal/mol); 8: ZINC000001607536_sub (-7.5 kcal/mol); 9: ZINC000001608397_sub (-7.0 kcal/mol); 10: ZINC000003833872_sub (-7.4 kcal/mol); 11: ZINC000004655109_sub (-6.7 kcal/mol); 12: ZINC000014758732_sub (-6.5 kcal/mol); 13: ZINC000014820468_sub (-6.4 kcal/mol); 14: ZINC000028865986_sim (-7.7 kcal/mol); 15: ZINC000049601467_sub (-4.6 kcal/mol); 16: ZINC000138252870_sub (-6.3 kcal/mol).

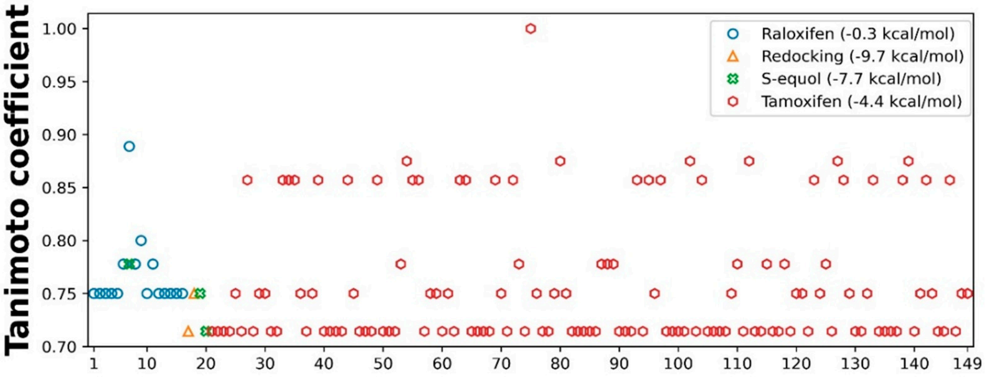


**Figure S4.** Selection of FDA compounds with interaction fingerprint similar to control compounds. Name of the compounds in the graph: 1: ZINC000000000346 (-8.0 kcal/mol); 2: ZINC000000000449 (-8.2 kcal/mol); 3: ZINC000000601316 (-8.6 kcal/mol); 4: ZINC000000968326 (-8.1 kcal/mol); 5: ZINC000001530706 (-7.0 kcal/mol); 6: ZINC000001548097 (-6.2 kcal/mol); 7: ZINC000001550766 (-6.7 kcal/mol); 8: ZINC000003785276 (-7.3 kcal/mol); 9: ZINC000003802417 (-5.6 kcal/mol); 10: ZINC000003927200 (-4.8 kcal/mol); 11: ZINC000012468792 (-6.2 kcal/mol); 12: ZINC000019632706 (-5.6 kcal/mol); 13: ZINC000022065398 (-7.1 kcal/mol); 14: ZINC000030691763 (-4.8 kcal/mol); 15: ZINC000085205451 (-6.6 kcal/mol); 16: ZINC000100036536 (-9.0 kcal/mol); 17: ZINC000000002272 (-9.1 kcal/mol); 18: ZINC000118912393 (-10.2 kcal/mol); 19: ZINC000000056652 (-7.0 kcal/mol); 20: ZINC000043207851 (-8.2 kcal/mol); 21: ZINC000000000347 (-5.4 kcal/mol); 22: ZINC000000000506 (-7.3 kcal/mol); 23: ZINC000000000655 (-8.0 kcal/mol); 24: ZINC000000001587 (-8.0 kcal/mol); 25: ZINC000000001982 (-7.3 kcal/mol); 26: ZINC000000002688 (-9.8 kcal/mol); 27: ZINC000000004778 (-8.2 kcal/mol); 28: ZINC000000004893 (-8.9 kcal/mol); 29: ZINC000000006251 (-7.5 kcal/mol); 30: ZINC000000011012 (-8.6 kcal/mol); 31: ZINC000000020248 (-7.5 kcal/mol); 32: ZINC000000020250 (-8.7 kcal/mol); 33: ZINC000000056556 (-7.8 kcal/mol); 34: ZINC000000057001 (-5.8 kcal/mol); 35: ZINC000000057062 (-6.3 kcal/mol); 36: ZINC000000120319 (-7.7 kcal/mol); 37: ZINC000000537752 (-6.5 kcal/mol); 38: ZINC000000538312 (-5.3 kcal/mol); 39: ZINC000000538483 (-7.3 kcal/mol); 40: ZINC000000538627 (-6.3 kcal/mol); 41: ZINC000000584092 (-5.8 kcal/mol); 42: ZINC000000591993 (-5.8 kcal/mol); 43: ZINC000000607939 (-6.0 kcal/mol); 44: ZINC000000607986 (-7.2 kcal/mol); 45: ZINC000000608382 (-5.5 kcal/mol); 46: ZINC000000896560 (-5.5 kcal/mol); 47: ZINC000000897085 (-9.4 kcal/mol); 48: ZINC000000897240 (-7.4 kcal/mol); 49: ZINC000000968273 (-8.6 kcal/mol); 50: ZINC000000968274 (-9.1 kcal/mol); 51: ZINC000000968275 (-9.0 kcal/mol); 52: ZINC000000968303 (-6.7 kcal/mol); 53: ZINC000000968336 (-8.3 kcal/mol); 54: ZINC000001530575 (-7.7 kcal/mol); 55: ZINC000001530579 (-6.5 kcal/mol); 56: ZINC000001530580 (-6.5 kcal/mol); 57: ZINC000001530613 (-7.3 kcal/mol); 58: ZINC000001530617 (-6.1 kcal/mol); 59: ZINC000001530618 (-6.5 kcal/mol); 60: ZINC000001530625 (-8.9 kcal/mol); 61: ZINC000001530689 (-5.0 kcal/mol); 62: ZINC000001530695 (-5.1 kcal/mol); 63: ZINC000001530752 (-6.9 kcal/mol); 64: ZINC000001530761 (-6.4 kcal/mol); 65: ZINC000001530814 (-9.0 kcal/mol); 66: ZINC000001530816 (-7.2 kcal/mol); 67: ZINC000001530817 (-7.2 kcal/mol); 68: ZINC000001530929 (-6.6 kcal/mol); 69: ZINC000001530939 (-7.0 kcal/mol); 70: ZINC000001530951 (-6.5 kcal/mol); 71: ZINC000001530975 (-8.4 kcal/mol); 72: ZINC000001530977 (-9.8 kcal/mol); 73: ZINC000001534965 (-5.9 kcal/mol); 74: ZINC000001542392 (-5.1 kcal/mol); 75: ZINC000001550499 (-10.0 kcal/mol); 76: ZINC000001554010 (-7.5 kcal/mol); 77: ZINC000001850374 (-7.7 kcal/mol); 78: ZINC000001850376 (-7.3 kcal/mol); 79: ZINC000001851149 (-6.3 kcal/mol); 80: ZINC000001886617 (-7.4 kcal/mol); 81: ZINC000001996117 (-4.8 kcal/mol); 82: ZINC000002016257 (-6.7 kcal/mol); 83: ZINC000002016258 (-6.8 kcal/mol); 84: ZINC000003794601 (-7.7 kcal/mol); 85: ZINC000003795819 (-9.1 kcal/mol); 86: ZINC000003798757 (-5.8 kcal/mol); 87: ZINC000003799072 (-6.6 kcal/mol); 88: ZINC000003807172 (-4.7 kcal/mol); 89: ZINC000003810860 (-9.2 kcal/mol); 90: ZINC000003812974 (-6.9 kcal/mol); 91: ZINC000003812988 (-6.5 kcal/mol); 92: ZINC000003812989 (-7.0 kcal/mol); 93: ZINC000003813088 (-6.9 kcal/mol); 94: ZINC000003830339 (-7.6 kcal/mol); 95: ZINC000003830713 (-7.1 kcal/mol); 96: ZINC000003831138 (-6.1 kcal/mol); 97: ZINC000003831165 (-7.1 kcal/mol); 98: ZINC000003831405 (-7.8 kcal/mol); 99: ZINC000003872520 (-5.6 kcal/mol); 100: ZINC000003872738 (-7.3 kcal/mol); 101: ZINC000003874950 (-6.9 kcal/mol); 102: ZINC000003875980 (-5.7 kcal/mol); 103: ZINC000003914808 (-5.2 kcal/mol); 104: ZINC000003929508 (-6.5 kcal/mol); 105: ZINC000003956788 (-8.0

kcal/mol); 106: ZINC000003972949 (-7.1 kcal/mol); 107: ZINC000003979899 (-6.3 kcal/mol); 108: ZINC000004217732 (-7.0 kcal/mol); 109: ZINC000004474414 (-4.6 kcal/mol); 110: ZINC000004474460 (-7.0 kcal/mol); 111: ZINC000004618208 (-7.4 kcal/mol); 112: ZINC000004629876 (-4.9 kcal/mol); 113: ZINC000004654889 (-7.4 kcal/mol); 114: ZINC000004676424 (-6.5 kcal/mol); 115: ZINC000004978673 (-6.9 kcal/mol); 116: ZINC000006094354 (-9.0 kcal/mol); 117: ZINC000006845963 (-7.3 kcal/mol); 118: ZINC000006920384 (-6.4 kcal/mol); 119: ZINC000007997897 (-9.7 kcal/mol); 120: ZINC000008214703 (-6.7 kcal/mol); 121: ZINC000008220878 (-6.2 kcal/mol); 122: ZINC000009212654 (-8.4 kcal/mol); 123: ZINC000011680943 (-7.2 kcal/mol); 124: ZINC000012404516 (-4.8 kcal/mol); 125: ZINC000012484958 (-8.8 kcal/mol); 126: ZINC000012503099 (-8.6 kcal/mol); 127: ZINC000012661824 (-8.3 kcal/mol); 128: ZINC000013973998 (-7.2 kcal/mol); 129: ZINC000014881137 (-6.3 kcal/mol); 130: ZINC000019156872 (-8.8 kcal/mol); 131: ZINC000019632614 (-4.7 kcal/mol); 132: ZINC000019632670 (-5.1 kcal/mol); 133: ZINC000033943508 (-8.8 kcal/mol); 134: ZINC000035801098 (-6.4 kcal/mol); 135: ZINC000035999642 (-7.6 kcal/mol); 136: ZINC000053084692 (-7.9 kcal/mol); 137: ZINC000084589076 (-9.0 kcal/mol); 138: ZINC000084758479 (-5.7 kcal/mol); 139: ZINC000096014710 (-8.2 kcal/mol); 140: ZINC000100001918 (-8.1 kcal/mol); 141: ZINC000100001965 (-5.9 kcal/mol); 142: ZINC000100006264 (-8.8 kcal/mol); 143: ZINC000100009278 (-7.8 kcal/mol); 144: ZINC000100018594 (-7.0 kcal/mol); 145: ZINC000100018854 (-5.4 kcal/mol); 146: ZINC000100296828 (-8.0 kcal/mol); 147: ZINC000101489663 (-7.7 kcal/mol); 148: ZINC000116473771 (-4.7 kcal/mol); 149: ZINC000410428674 (-4.5 kcal/mol).

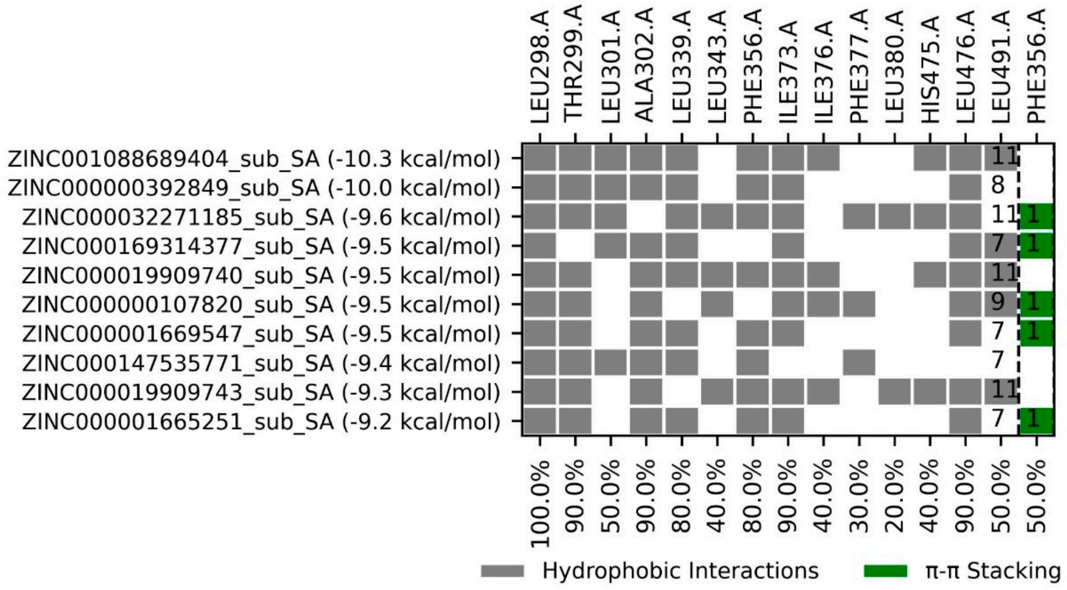


**Figure S5.** Interaction profile of the top ten compounds derived from scaffold A, docked to the active site of ERβ. The number at the end of each type of interaction is the total number of interactions for each compound.

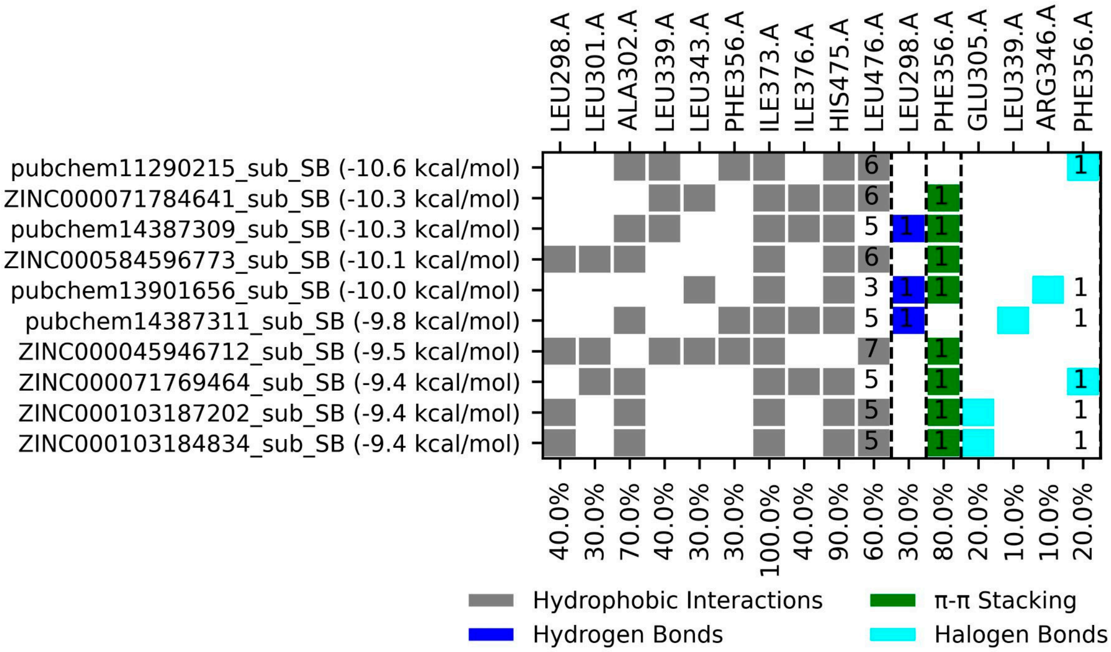


**Figure S6.** Interactions of the top ten scaffold B compounds docked at the active site of the ERβ.

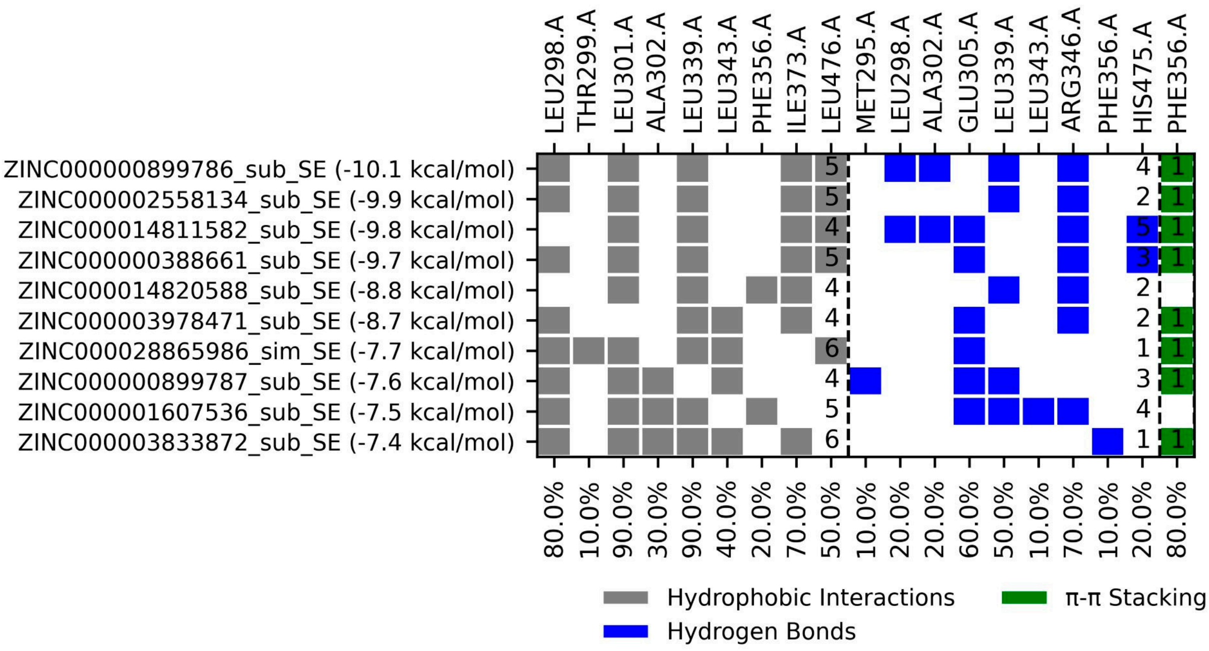


**Figure S7.** Interactions of the top ten compounds of s-equol analogs on the active site of ERβ.

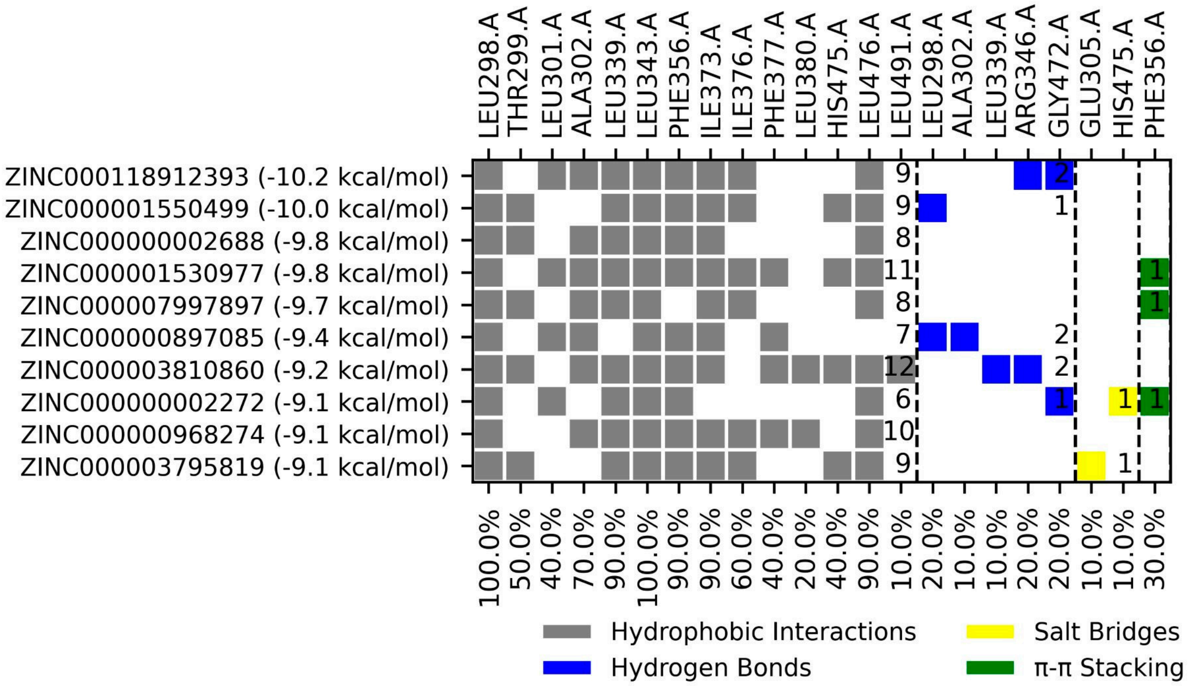


**Figure S8.** The top ten FDA compounds docked to the ERβ active site.

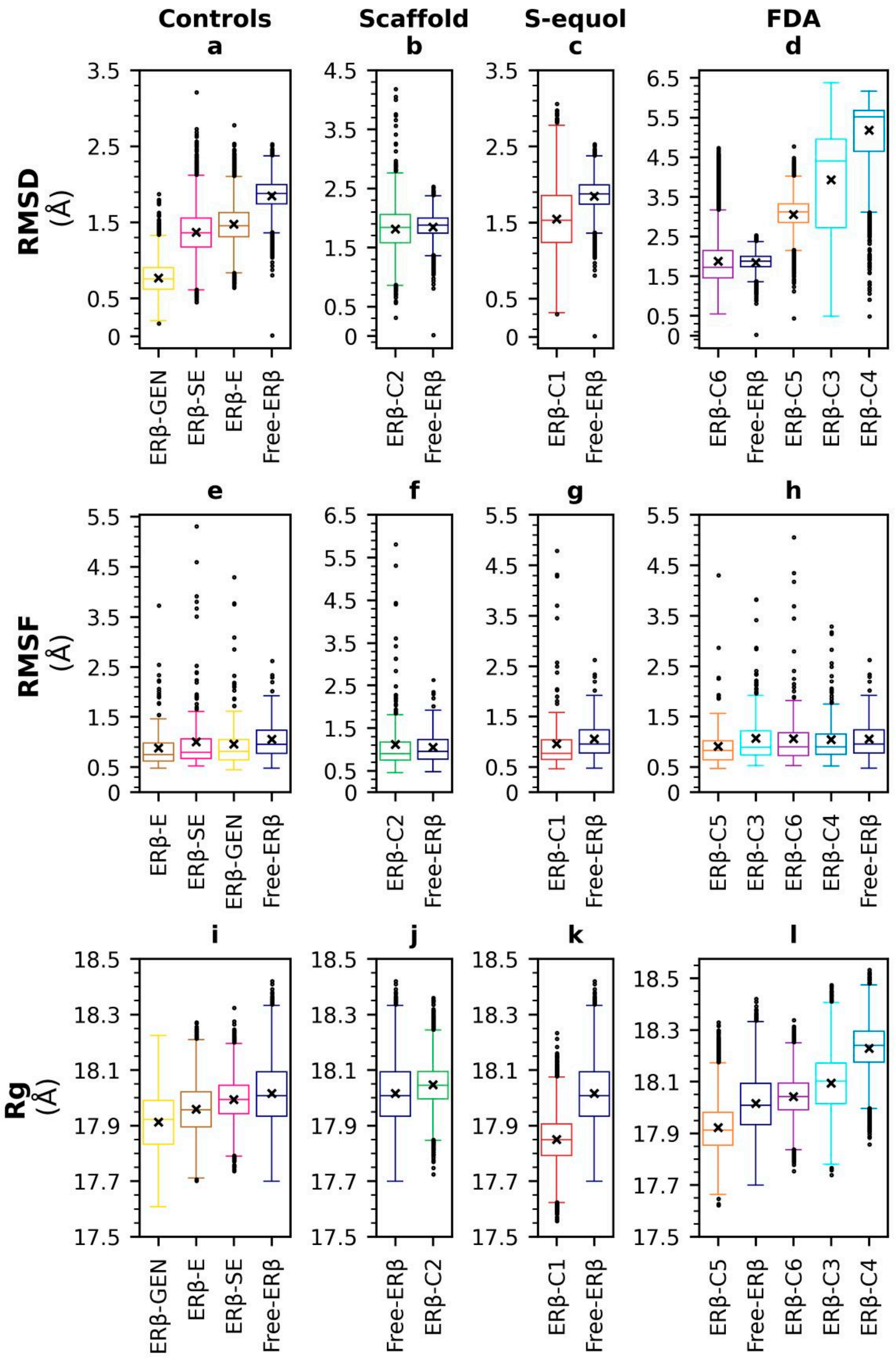


**Figure S9.** Box plot of RMSD (a-d), RMSF (e-h) and Rg (i-l). The graphs are ordered according to the median, the average is represented by an "x", the points denote outliers.

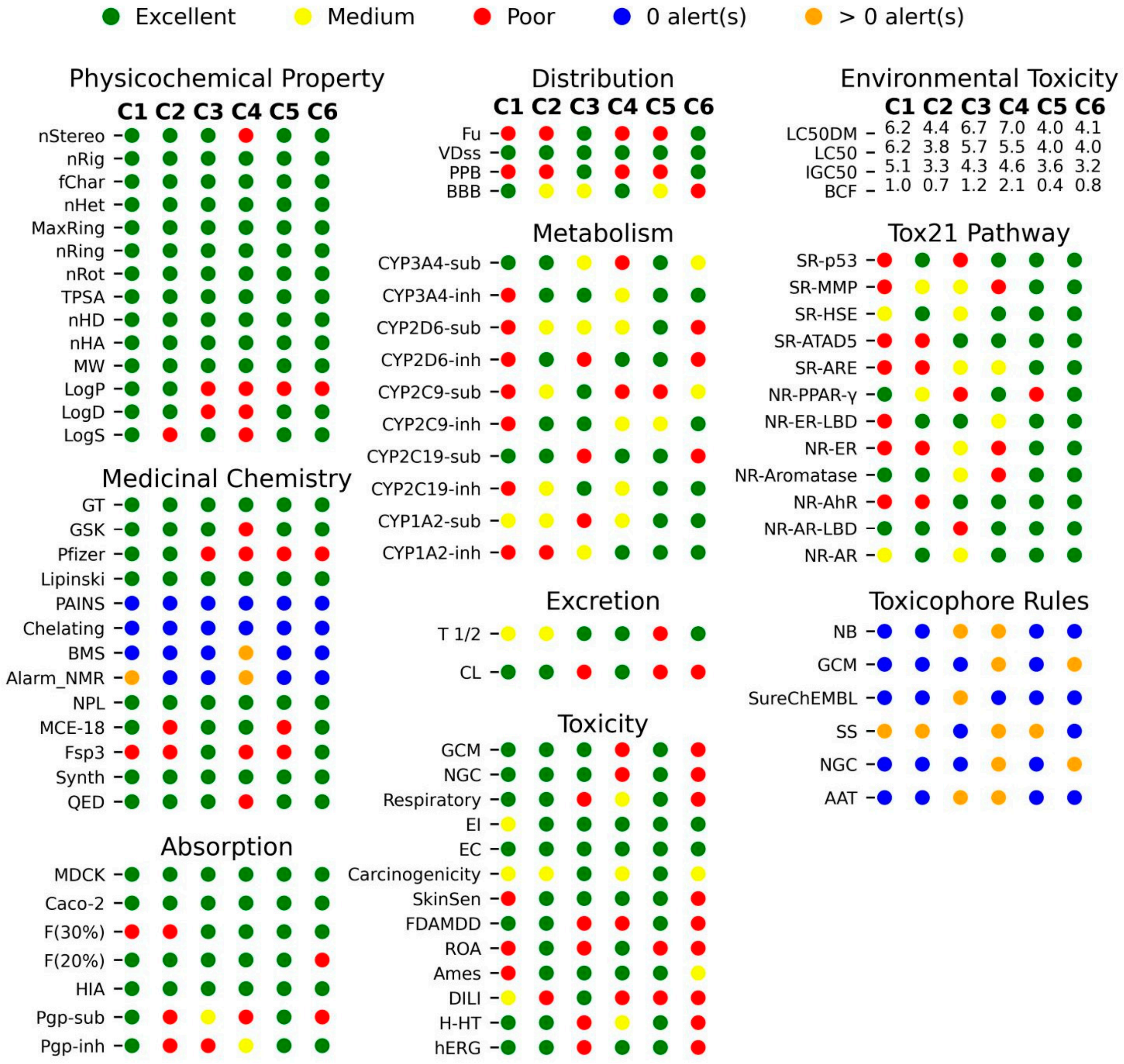


**Figure S10.** Evaluation ADMET of compounds C1-6.
